# Effects of 8-week plyometric training program on balance, reaction time, and attention on elite adolescent female taekwondo athletes

**DOI:** 10.3389/fspor.2026.1838014

**Published:** 2026-07-15

**Authors:** Harun Genç, Mustafa Türkmen, İrem Tekin, Ali Erdem Cigerci, Veli Volkan Gürses, Oktay Kızar, Mehmet Şerif Ökmen, İsmail Polatcan, Serhat Doğu Gündoğdu

**Affiliations:** 1 Bingöl University, Bingöl, Türkiye; 2 TC Mardin Artuklu Universitesi, Mardin, Türkiye; 3 Kastamonu Universitesi, Kastamonu, Türkiye; 4 Bandirma Onyedi Eylul Universitesi, Bandırma, Türkiye; 5 Munzur Universitesi, Tunceli, Türkiye

**Keywords:** attention, balance, female taekwondo athletes, plyometric, reaction time

## Abstract

**Introduction:**

Taekwondo is a complex combat sport requiring high levels of strength, balance, reaction time, attention, agility, and endurance for effective performance under rapid offensive and defensive conditions. This study aimed to investigate the effects of an 8-week plyometric training program on static balance (SB), dynamic balance (DB), reaction time (RT), and attention (AT) in adolescent female taekwondo athletes.

**Methods:**

Twenty female athletes (age: 14.85 ± 1.53 years; height: 162.27 ± 7.12 cm; body mass: 56.69 ± 6.45 kg) voluntarily participated in the study at Mardin Halit Demir Martial Arts Club. Participants were randomly assigned to either an experimental group (EG, *n* = 10) or a control group (CG, *n* = 10). The EG performed plyometric training twice weekly for 8 weeks in addition to regular taekwondo training, while the CG continued only taekwondo training. Static balance was defined as the primary outcome, whereas dynamic balance, reaction time, and attention were considered secondary outcomes. A 2 × 2 repeated-measures analysis of variance (group × time) was used to examine intervention effects.

**Results:**

Significant group × time interactions were observed for static balance and dynamic balance (*p* < 0.05), favoring the experimental group. However, no significant group × time interactions were found for reaction time or attention (*p* > 0.05).

**Discussion:**

These findings suggest that plyometric training may effectively improve balance performance in adolescent female taekwondo athletes, particularly static and dynamic balance. However, no significant effects were observed for reaction time or attention. Further studies with larger sample sizes are needed to clarify the potential effects of plyometric training on cognitive performance.

## Introduction

Taekwondo is a combat sport originating from South Korea that primarily involves striking techniques performed using both the upper and lower extremities. It has achieved considerable popularity on a global scale (1). The execution of techniques such as punches and kicks to the opponent's head or body requires athletes to perform rapid and explosive limb movements. Match analysis studies indicate that taekwondo athletes predominantly utilize lower extremity techniques to score points (2, 3).

In this context, lower limb power plays a critical role in performance, making plyometric training a particularly effective method for enhancing explosive strength and speed in taekwondo athletes. Plyometric exercises, characterized by the stretch-shortening cycle, are widely recognized for improving power output, agility, and neuromuscular efficiency across various sports disciplines (4, 5). In combat sports such as taekwondo, these qualities are essential for executing rapid attacks, defensive actions, and directional changes.

Evidence from combat sports and youth athletic populations suggests that plyometric training may improve dynamic balance, agility, and neuromuscular performance (6, 7). In particular, studies on adolescent taekwondo and similar combat sport athletes have reported improvements in balance and movement efficiency following 6–8 week plyometric training interventions (8, 9).

Beyond physical performance, cognitive factors such as reaction time and attention also play a crucial role in taekwondo success. Athletes must process rapidly changing visual stimuli and respond with precise timing during combat situations (10). In this regard, emerging evidence suggests that plyometric training may also have indirect benefits on cognitive-motor performance by enhancing neuromuscular coordination and information processing speed. The adolescent period represents a crucial phase for physical growth and development. The implementation of suitable training programs during this phase can have a significant and enduring impact on an athlete's long-term performance and overall health (11). Therefore, integrating high-intensity training methods such as plyometrics during this period may provide meaningful performance adaptations when properly controlled.

Although previous research has extensively examined the effects of plyometric training in adult athletes, studies focusing on adolescent taekwondo athletes remain limited, particularly regarding cognitive variables such as attention and reaction time. Recent studies have begun to address this gap; however, findings remain limited and inconsistent, especially regarding combined physical and cognitive adaptations.

In the present study, static balance was predefined as the primary outcome measure because balance is considered a fundamental component of taekwondo performance and is highly dependent on lower-extremity neuromuscular function. Dynamic balance, reaction time, and attention were designated as secondary outcomes.

The principal inferential hypothesis of the study was that an eight-week plyometric training program would produce significantly greater improvements in static balance compared with regular taekwondo training alone. Secondary hypotheses were that plyometric training would also improve dynamic balance, reaction time, and attention performance.

The present study aimed to evaluate the effects of an eight-week plyometric training program on static balance, dynamic balance, reaction time, and attention in elite adolescent female taekwondo athletes. Furthermore, the study aimed to address an important gap in the literature by simultaneously examining motor and cognitive adaptations following plyometric training in elite adolescent female taekwondo athletes.

## Material & methods

### Participants

Twenty female adolescent taekwondo athletes voluntarily participated in this study. Participants were randomly assigned to an experimental group (EG, *n* = 10) and a control group (CG, *n* = 10) using a simple randomization procedure. All participants were actively training at the Mardin Halit Demir Martial Arts Club.

An *a priori* power analysis was conducted using G*Power software (version 3.1.9.2, Heinrich-Heine-Universität Düsseldorf, Germany) to determine the minimum required sample size. Based on a repeated measures ANOVA design (within–between interaction), an effect size of 0.25, an alpha level of 0.05, and a statistical power of 0.80 were assumed. The analysis indicated that a minimum sample size of approximately 16–20 participants was required. Therefore, the final sample size of 20 participants was considered adequate to achieve sufficient statistical power for the present study design. The selected effect size was justified based on previous studies investigating plyometric training effects on balance, reaction time, and neuromuscular performance in adolescent athletes.

### Eligibility criteria

Participants were required to meet the following inclusion criteria: (i) female taekwondo athletes aged 13–16 years, (ii) at least 3 years of systematic training experience, (iii) licensed athletes actively training in a taekwondo club, (iv) regular training attendance (≥3 sessions per week), and (v) written informed consent from both athletes and their parents or legal guardians.

“Elite” status was operationally defined as active participation in official national-level taekwondo competitions sanctioned by the national federation, possession of at least a black belt rank (1st dan or higher), and prior experience in regional and/or national championships. This ensured that all participants represented competitively experienced and systematically trained adolescent athletes.

Exclusion criteria included: (i) any acute or chronic orthopedic or neurological disorder, (ii) history of musculoskeletal injury within the previous 6 months, (iii) any medical condition limiting high-intensity physical activity, (iv) absence from training sessions during the intervention period, and (v) incomplete pre- or post-intervention assessments.

Participants who did not meet these criteria were excluded to ensure sample homogeneity and to minimize potential confounding factors.

Table 1 presents the descriptive characteristics of the participants. The study included 20 participants. The mean age was 14.85 ± 1.53 years. The mean body height was 162.27 ± 7.12 cm, and the mean body weight was 56.69 ± 6.45 kg. The mean body mass index (BMI) was 21.56 ± 2.67 kg/m^2^.

**Table 1:** Descriptive statistics of participants.

Variables	*N*	Mean	SD	Minimum	Maximum
Age (year)	20	14.85	1.53	13.00	16.00
Height (m)	20	162.27	7.12	150.00	180.50
BW (kg)	20	56.69	6.45	47.50	70.30
BMI (kg/m^2^)	20	21.56	2.67	18.22	27.56

BW, body weight; BMI, body mass index.

### Ethical considerations

This study was conducted in accordance with the Declaration of Helsinki and approved by the Ethics Committee of the Institute of Health Sciences, Mardin Artuklu University (approval number: 2024/3–37; date: 05/03/2024).

### Experimental design and procedures

A randomized pre–post experimental design was employed. Participants were randomly assigned to either an experimental group (EG) or a control group (CG). Static balance, dynamic balance, reaction time, and attention were assessed in both groups before and after the intervention. Prior to testing sessions, all participants performed a standardized warm-up consisting of 15 min of light jogging followed by 10 min of stretching. After a sufficient rest period, pre- and post-test measurements were conducted under identical conditions. Following the pre-tests, the EG completed an 8-week plyometric training program consisting of 60-minute sessions performed twice weekly on non-consecutive days in addition to regular taekwondo training, while the CG continued only their regular taekwondo training. The plyometric training protocol included a 20-minute warm-up, main exercises, and a 15-minute cool-down period. Progressive overload was applied by systematically increasing sets and repetitions throughout the intervention period. Participants were instructed to avoid strenuous physical activity for 24 h before testing, to maintain normal sleep patterns, and not to consume any performance-enhancing medication during the study period.

Each plyometric training session was conducted in a circuit training format consisting of 15 exercise stations. Participants performed all exercises in a predetermined fixed order as presented in Table 2. Sessions began with a standardized warm-up including 5–10 min of light aerobic activity and dynamic stretching.

**Table 2:** 8-week plyometric training program for participants.

Tuesday—Thursday	Weeks 1–2	Weeks 3–4	Weeks 5–6	Weeks 7–8
Jump Rope+All Exercises	5 min	10 min	15 min	20 min
Sets and Repetitions	1 × 10	2 × 10	3 × 10	3 × 15
Rest Between Repetitions	30 Sec	30 Sec	30 Sec	30 Sec
Rest Between Sets	1.5 min	1.5 min	1.5 min	1.5 min
Exercise Stations				
1: Jump Rope 2: Single-leg jumps using arms 3: Single-leg jumps without using arms 4: Double-leg forward hop 5: Single-leg forward hop 6: Hexagonal work 7: Lateral jump over an obstacle 8: Direction change with long jump Station	9: Double-leg jump from the ground to the box 10: Forward jump over a cone 11: Double-leg jump from the box to the ground 12: Push body upward by changing feet 13: Double-leg jump with knees pulled to the chest 14: Double-leg jump without using arms 15: Double-leg jump using arms			

The circuit included the following exercises in sequence: jump rope, single-leg jumps with arm swing, single-leg jumps without arm swing, double-leg forward hops, single-leg forward hops, hexagonal agility drill, lateral jumps over an obstacle, directional change long jumps, double-leg box jumps, box drop jumps, cone jumps, double-leg jumps with foot alternation, double-leg tuck jumps, double-leg jumps without arm involvement, and double-leg jumps with arm involvement.

Each exercise station was performed for the prescribed repetitions and sets, with 30 s of rest between repetitions and 1.5 min of rest between sets.

The training load was progressively increased every two weeks by systematically increasing jump rope duration and manipulating sets and repetitions as shown in Table 2. Although total jump count was not separately quantified, progressive overload was ensured through structured external volume manipulation.

Internal training load (e.g., session rating of perceived exertion or heart rate monitoring) was not recorded due to practical constraints; however, all sessions were supervised by the same qualified coach to ensure standardized training intensity and execution.

All participants in the experimental group completed the full intervention, resulting in a 100% compliance rate. No injuries or dropouts occurred during the study period. Recovery between repetitions and sets was standardized using fixed rest intervals, and no additional recovery monitoring tools were applied.

Each session concluded with a cool-down period consisting of light stretching exercises.

### Measurements

#### Dynamic balance measurement

Dynamic balance measurements were performed using the Gymstick Performance Balance Board (Gymstick International Oy, Finland), a laminated hardwood balance platform with a non-slip surface and adjustable height designed to improve balance, coordination, and core stability (12). The participant was instructed to stand on the balance board with both feet in an upright position while keeping the arms parallel to the body. The balance measurement was performed in 3 repetitions. Each repetition consisted of 30 s of balance exercise followed by 10 s of rest. The data were recorded as the average of the repetitions. The test was terminated and the protocol was repeated if a participant experienced more than 5 instances of loss of balance during the test.

#### Static balance measurement

The assessor tries to maintain balance on one leg (on a beam 50 cm long, 4 cm high, and 3 cm wide), the arm on the same side is raised, the other lower limb is flexed, raised back and caught by the hand on the same side. We record the number of attempts/failures required to accumulate 60 s of holding the position (13).

#### Reaction time measurement

Prior to the actual testing, all participants completed a familiarization trial in order to become accustomed to the testing procedures and to minimize any learning effect. Reaction time was assessed using a computer-based visual reaction time test (Human Benchmark reaction time test, https://humanbenchmark.com/tests/reactiontime). Participants were instructed to respond as quickly as possible by clicking on the screen when the visual stimulus changed from red to green. The test was administered on a touch-screen computer, and reaction time was automatically recorded in milliseconds when the participant responded. Each participant completed five trials, and the mean value of these trials was used for statistical analysis. Lower scores indicated faster and better reaction time performance (14).

The Human Benchmark reaction time test is widely used in sport science and cognitive research as a practical tool for assessing simple visual reaction time and has demonstrated acceptable reliability for group-level comparisons. Although the system provides millisecond-level output, a small measurement variability (approximately 10–50 ms) may occur due to inherent limitations of browser-based timing systems. However, because this variability is expected to be systematic across participants in a randomized design, it is unlikely to bias between-group comparisons. In addition, while laboratory-based systems may provide higher temporal precision, this web-based test offers a practical and ecologically relevant assessment of sport-specific simple reaction time under accessible testing conditions.

#### Attention assessment

Prior to the actual testing, all participants completed a familiarization trial to become accustomed to the testing procedures and to minimize any learning effect. Attention performance was assessed using a computer-based visual attention task (Human Benchmark online platform). Participants were instructed to respond as quickly as possible by tapping targets that appeared at different locations on the screen. The task consisted of 30 target responses per trial, and three trials were completed for each participant. Performance was recorded in milliseconds, and the mean of the three trials was used for statistical analysis. Lower scores indicated better attention performance. Although the task provides millisecond-level output, a small measurement variability (approximately 10–50 ms) may occur due to inherent limitations of browser-based timing systems (15).

### Statistical analysis

All statistical analyses were performed using SPSS Statistics 27.0 (IBM, USA). Descriptive statistics were presented as means, standard deviations (±SD), and 95% confidence intervals (CI) for all continuous variables to provide estimation-based interpretation of the results.

Static balance was defined as the primary outcome, while dynamic balance, reaction time, and attention were considered secondary outcomes. The primary inferential hypothesis focused on expected greater improvements in static balance following plyometric training compared to regular taekwondo training.

Normality of data distribution was assessed using the Shapiro–Wilk test, and homogeneity of variance was evaluated using Levene's test. The assumptions for repeated measures analysis were examined prior to inferential testing. Given the 2 × 2 design (time×group), sphericity was not a concern.

A 2 × 2 repeated measures analysis of variance (ANOVA) was used to examine the main effects of time (pre-test vs. post-test), group (plyometric training vs. control), and the time×group interaction for all dependent variables. When a significant interaction effect was observed, pairwise comparisons were conducted using Bonferroni adjustment to control for Type I error inflation.

To complement null hypothesis testing, effect sizes were calculated using partial eta-squared (*η*^2^p), interpreted as small (0.01), medium (0.06), and large (0.14 or greater). In addition, percentage change (Δ%) values were calculated using the formula: (post-test−pre-test)/pre-test×100 to describe the magnitude of performance changes.

Confidence intervals (95% CI) were used alongside *p*-values and effect sizes to support interpretation of group differences and interaction effects, in line with contemporary reporting recommendations emphasizing estimation-based statistics.

The interpretation of time×group interactions was based not only on statistical significance but also on the direction and magnitude of change between groups over time. This approach allowed a more comprehensive evaluation of training-related adaptations.

An *a priori* power analysis was conducted using G*Power (version 3.1.9.2, Germany), indicating that the study achieved 80% statistical power with an effect size range of 0.25–0.39 and an alpha level of 0.05, confirming adequate sample size for detecting medium-to-large effects.

## Results

A total of 20 athletes participated in the study, with demographic characteristics summarized in Table 1.

Table 3 shows the results of the two-way repeated measures ANOVA. A significant time×group interaction was observed for dynamic balance (*F* = 5.225, *p* = 0.035, *η*^2*p*^ = 0.225) and static balance (*F* = 10.441, *p* = 0.005, *η*^2*p*^ = 0.367), while no significant interaction was found for reaction time (*F* = 2.801, *p* = 0.112, *η*^2*p*^ = 0.135) or attention (*F* = 1.590, *p* = 0.223, *η*^2*p*^ = 0.081). Significant main effects of time were observed for static balance (*F* = 4.503, *p* = 0.048, *η*^2*p*^ = 0.200) and reaction time (*F* = 5.592, *p* = 0.029, *η*^2*p*^ = 0.237), whereas no significant time effects were found for dynamic balance or attention. For the group effect, significant differences were identified for reaction time (*F* = 14.990, *p* = 0.001, *η*^2*p*^ = 0.454) and attention (*F* = 10.239, *p* = 0.005, *η*^2*p*^ = 0.363), with no significant differences for dynamic or static balance.

**Table 3:** Pre- and post-test results for balance, reaction time, and attention variables in experimental and control groups.

Results	Tests	Experimental (*n*:10) x¯ ± sd	Control (*n*:10) x¯ ± sd	Δ% Change	F Sig. ES	Time	Group	Time × Group
Dynamic Balance (rep)	Pre-test	47.00 ± 10.75	50.36 ± 10.67	−13.76%	F p Partial *η*^2^	0.009 0.926 0.00	3.63 0.073 0.168	5.225 0.035* 0.225
	Post-test	40.53 ± 13.18	37.43 ± 12.44	−25.67%				
Static Balance (rep)	Pre-test	9.30 ± 2.95	9.90 ± 2.99	−9.68%	F p Partial η^2^	5.224 0.035* 0.225	4.503 0.048* 0.200	10.441 0.005** 0.367
	Post-test	8.40 ± 3.92	4.90 ± 3.69	−50.50%				
Reaction Time (sec)	Pre-test	391.30 ± 79.30	353.40 ± 38.56	−6.21%	F p Partial η^2^	14.990 0.001* 0.454	5.592 <0.029* 0.237	2.801 0.112 0.135
	Post-test	367.00 ± 69.59	292.10 ± 34.64	−17.35%				
Attention (ms)	Pre-test	597.40 ± 71.28	566.10 ± 96.53	−5.07%	F p Partial η^2^	10.239 <0.005** 0.363	3.166 <0.092 0.150	1.590 0.223 0.081
	Post-test	567.10 ± 63.39	496.40 ± 45.81	−12.31%				

*p < 0.05; **p < 0.001; sec, second; ES, effect size; Data are presented as mean  ± SD I; 2-way analysis of variance with repeated measures (time; group; group×time) was used to assess the statistical significance of 8 weeks plyometric training-related effects.

**Figure 1:**
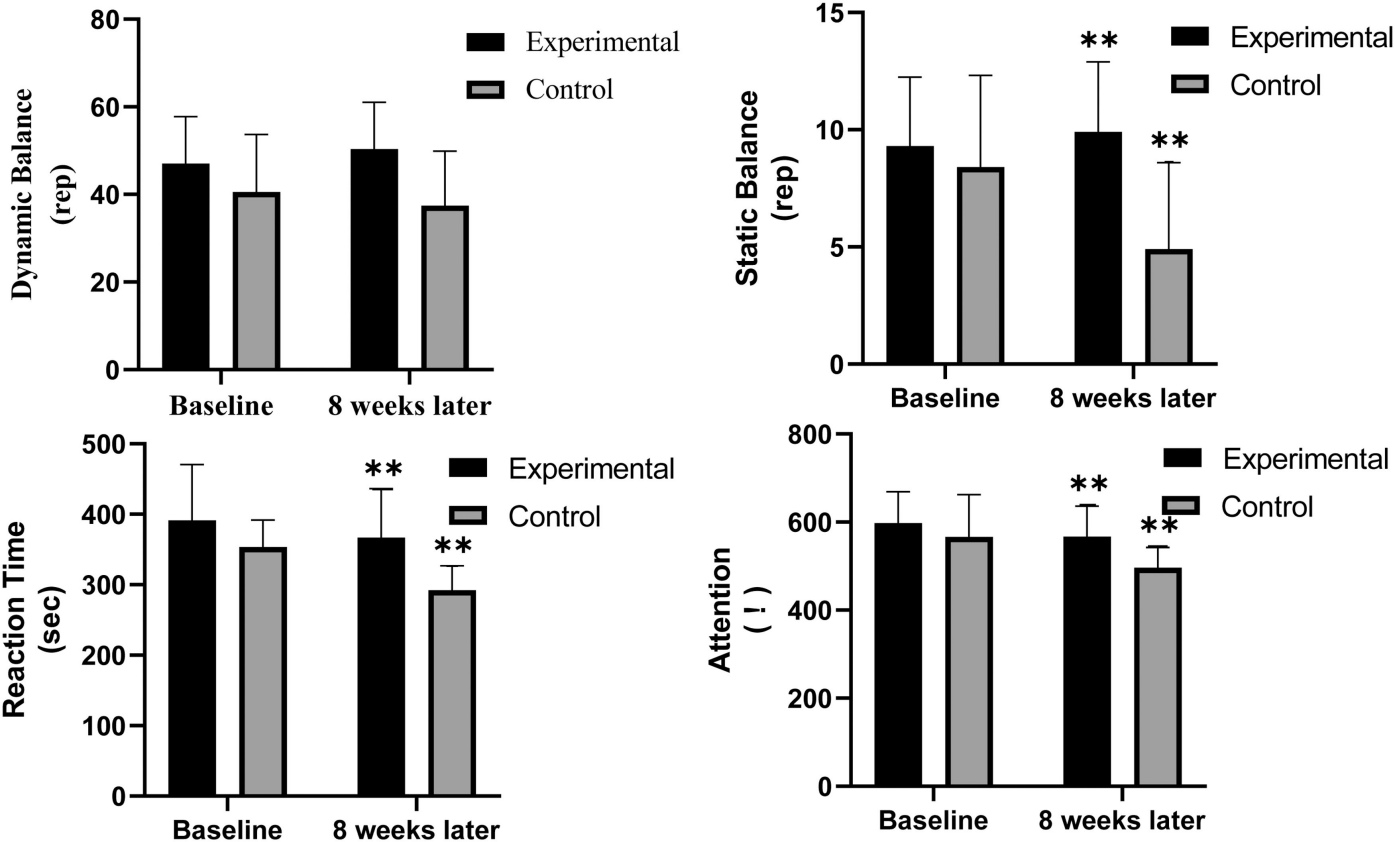
The figure shows pre- and post-intervention mean (SD) values for the experimental and control groups. The symbol * indicates *p* < 0.05 and ** indicates *p* < 0.01.

Figure 1 illustrates the study results. Static balance improved by 6.45% in the experimental group and by 41.66% in the control group after 8 weeks. Reaction time improved by 9.69% in the experimental group and by 20.40% in the control group at the end of the intervention period.

## Discussion

The present study examined the effects of an 8-week plyometric training program on static balance, dynamic balance, reaction time, and attention in elite adolescent female taekwondo athletes. A significant time×group interaction was found for static balance. These findings indicate that training-induced adaptations may differ across physical and cognitive performance domains depending on training specificity. The interpretation of these results is strengthened by the inclusion of 95% confidence intervals, which provide additional information regarding the precision and stability of the observed effects.

A significant time×group interaction was observed for static balance. Plyometric training appears to enhance static balance primarily through improvements in neuromuscular coordination and proprioceptive efficiency. The rapid stretch–shortening cycle characteristic of plyometric exercises may increase muscle spindle sensitivity and improve joint position sense, thereby contributing to more efficient postural control mechanisms. This interpretation is supported by previous studies reporting that plyometric interventions improve balance performance in youth athletes and individuals with functional instability (15–18). In combat sports such as taekwondo, where rapid postural adjustments are required, these adaptations may provide a performance advantage by enhancing the stability of the center of mass during dynamic movement transitions (19).

A significant time×group interaction was observed for dynamic balance, indicating that plyometric training elicited greater adaptations in dynamic postural control compared with traditional training. This improvement may be explained by task-specific neuromuscular adaptations associated with plyometric exercises. In particular, repeated exposure to rapid eccentric–concentric muscle actions may enhance intermuscular coordination and improve the efficiency of anticipatory postural adjustments. Such adaptations are critical for maintaining stability during movements requiring rapid postural control, such as changes of direction, which are frequently encountered in combat sports like taekwondo. Previous studies have also reported beneficial effects of short-term plyometric training interventions on dynamic balance and postural control (20, 21), suggesting that these improvements may be related to enhanced neuromuscular efficiency and sensorimotor integration. However, inconsistent findings have also been reported in the literature (22–24), and these discrepancies may be attributable to variations in training volume and intensity, participant characteristics, and the specificity of the balance assessment protocols employed.

Regarding reaction time and attention, reaction time demonstrated significant main effects of time and group, whereas attention showed a significant group effect without a significant time×group interaction. Although descriptive data suggested a greater improvement in reaction time in the control group, the lack of a significant interaction indicates that this finding should be interpreted cautiously. Reaction time improvements in taekwondo athletes may be explained by repeated exposure to sport-specific perceptual–cognitive demands that require rapid stimulus identification, continuous decision-making under time constraints, and fast motor response selection in unpredictable offensive and defensive situations. These processes are essential in combat sports, where performance depends on rapid information processing and execution under pressure. Previous studies have highlighted the importance of perceptual–cognitive skills in combat sport performance (25), while rapid motor response capacity has been identified as a key determinant of elite performance efficiency (26). In addition, faster reaction time has been associated with superior decision-making abilities in elite athletes, further emphasizing its relevance in high-performance contexts (27). Attention, as a fundamental cognitive component in precision and combat sports, contributes to sustained focus and information processing efficiency during performance (28). Although plyometric training has been reported to improve reaction time in other athletic populations such as badminton players (29), the absence of a significant interaction effect in the present study suggests that cognitive adaptations may be more strongly influenced by sport-specific training rather than plyometric training alone.

Interestingly, cognitive adaptations observed across both groups may be explained by training-induced neurocognitive plasticity associated with long-term sport participation. Regular engagement in taekwondo may stimulate cognitive processes such as sustained attention, working memory, and rapid information processing, which are continuously activated during training and competition. Such adaptations may occur even in the absence of structured cognitive training due to the inherently complex and variable nature of combat sports. Previous research has reported improvements in reaction time, rhythm, and cognitive performance following Life Kinetik training (30), which combines physical movement with simultaneous cognitive challenges to enhance neurocognitive efficiency. Similarly, cognitive-motor training interventions have been shown to improve attention and executive functions in adolescent athletes (31, 32), supporting the notion that integrated physical–cognitive demands can facilitate neurocognitive adaptation. These findings suggest that cognitive performance improvements in athletes may be more strongly influenced by the overall cognitive load of sport participation rather than isolated training modalities.

From a broader perspective, plyometric training appears to enhance both static and dynamic balance through neuromuscular and proprioceptive adaptations, whereas cognitive adaptations seem to be more strongly influenced by sport-specific training demands and overall cognitive load rather than plyometric training alone.

Finally, the relatively small sample size should be considered when interpreting the findings, as it may limit statistical power and generalizability. In addition, biological maturation status, including pubertal development and peak height velocity, was not assessed and may have influenced neuromuscular and cognitive adaptation responses, which should be acknowledged as a methodological limitation.

## Conclusions

In conclusion, the findings of the present study indicate that an 8-week plyometric training program integrated into taekwondo practice may contribute to improvements in static and dynamic balance in adolescent taekwondo athletes. Improvements in reaction time and attention were observed across the study period; however, no significant group×time interaction effects were identified for these variables. Therefore, the cognitive effects of plyometric training should be interpreted with caution and require further investigation. Future studies should consider training specificity, intervention duration, and methodological limitations when examining balance and cognitive outcomes in adolescent athletes.

## Data Availability

The raw data supporting the conclusions of this article will be made available by the authors, without undue reservation.
